# Supplementary material for: Gut microbiome alterations in canine idiopathic epilepsy: a pairwise case-control study
Source: Anim Microbiome. 2026 Jul 11;8:100. doi: 10.1186/s42523-026-00594-1 (PMC13644061; doi:10.1186/s42523-026-00594-1)
Supplement: Supplementary file 1 [file 42523_2026_594_MOESM1_ESM.pdf]

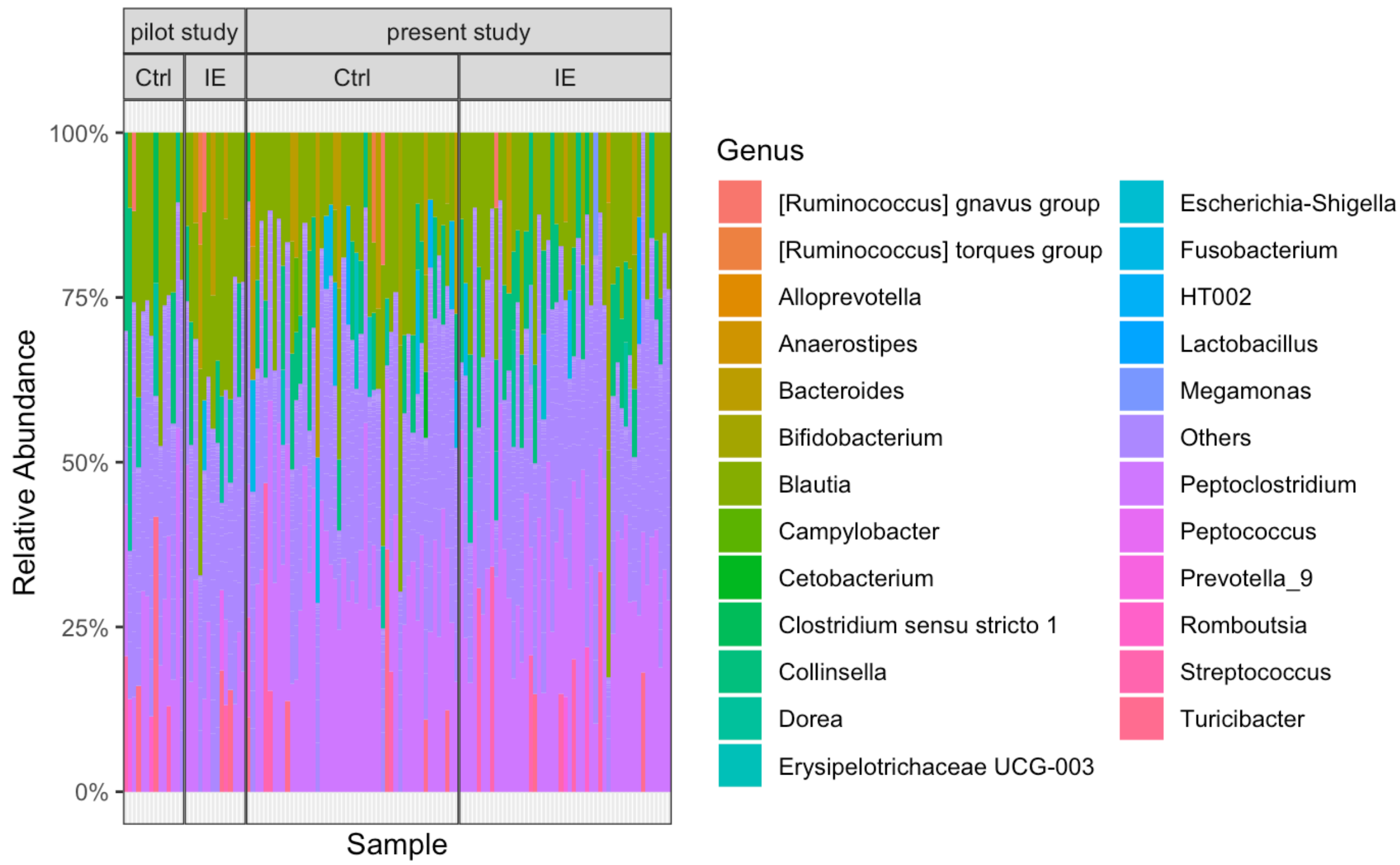

Figure S1: Relative abundances of bacterial genera in fecal samples from the pilot and present studies. Genera with relative abundance less than 10% were classified as Others.

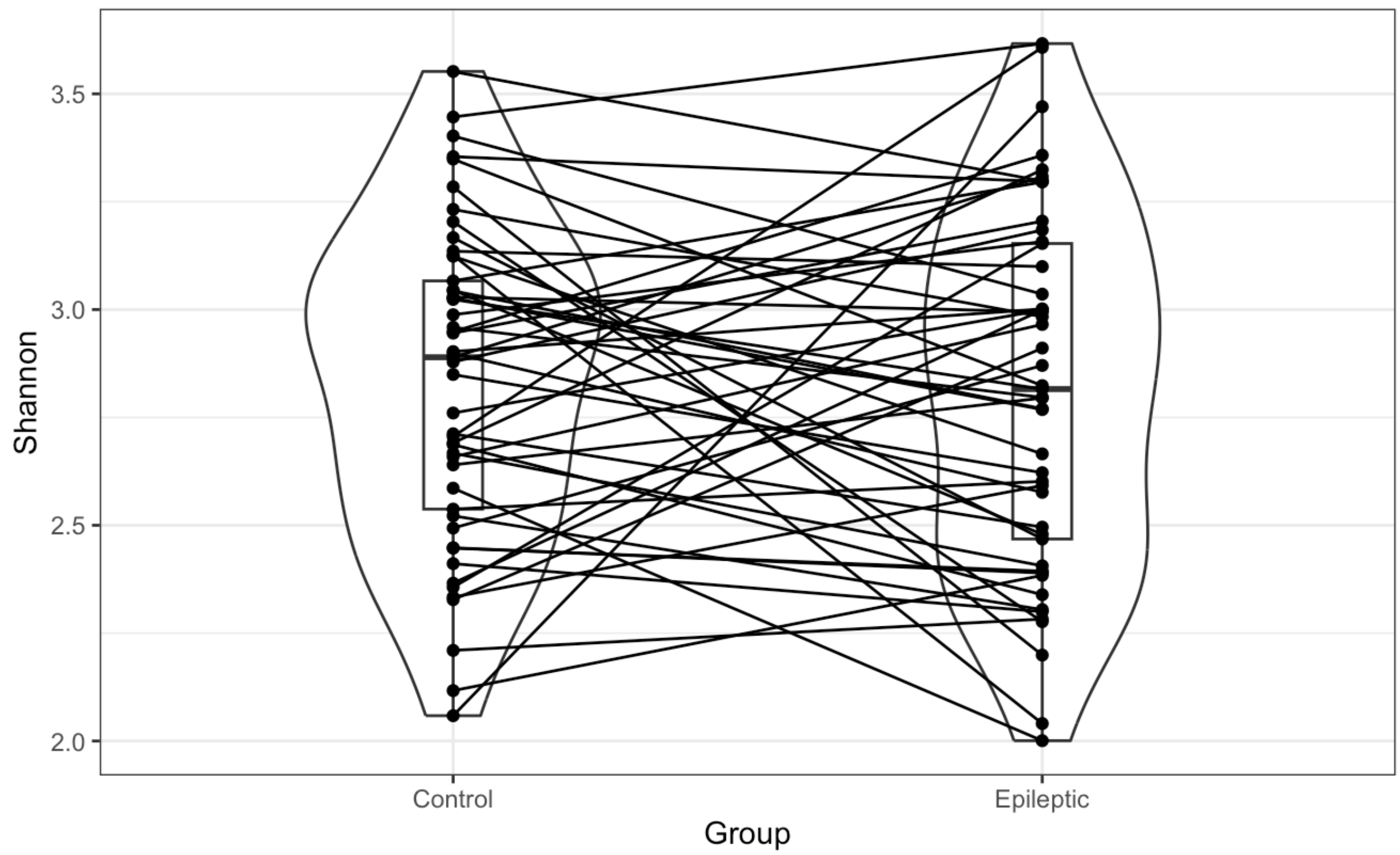

Figure S2: Shannon diversity index comparison between IE and control dogs. Each point represents one dog. Lines connect paired dogs from the same household.

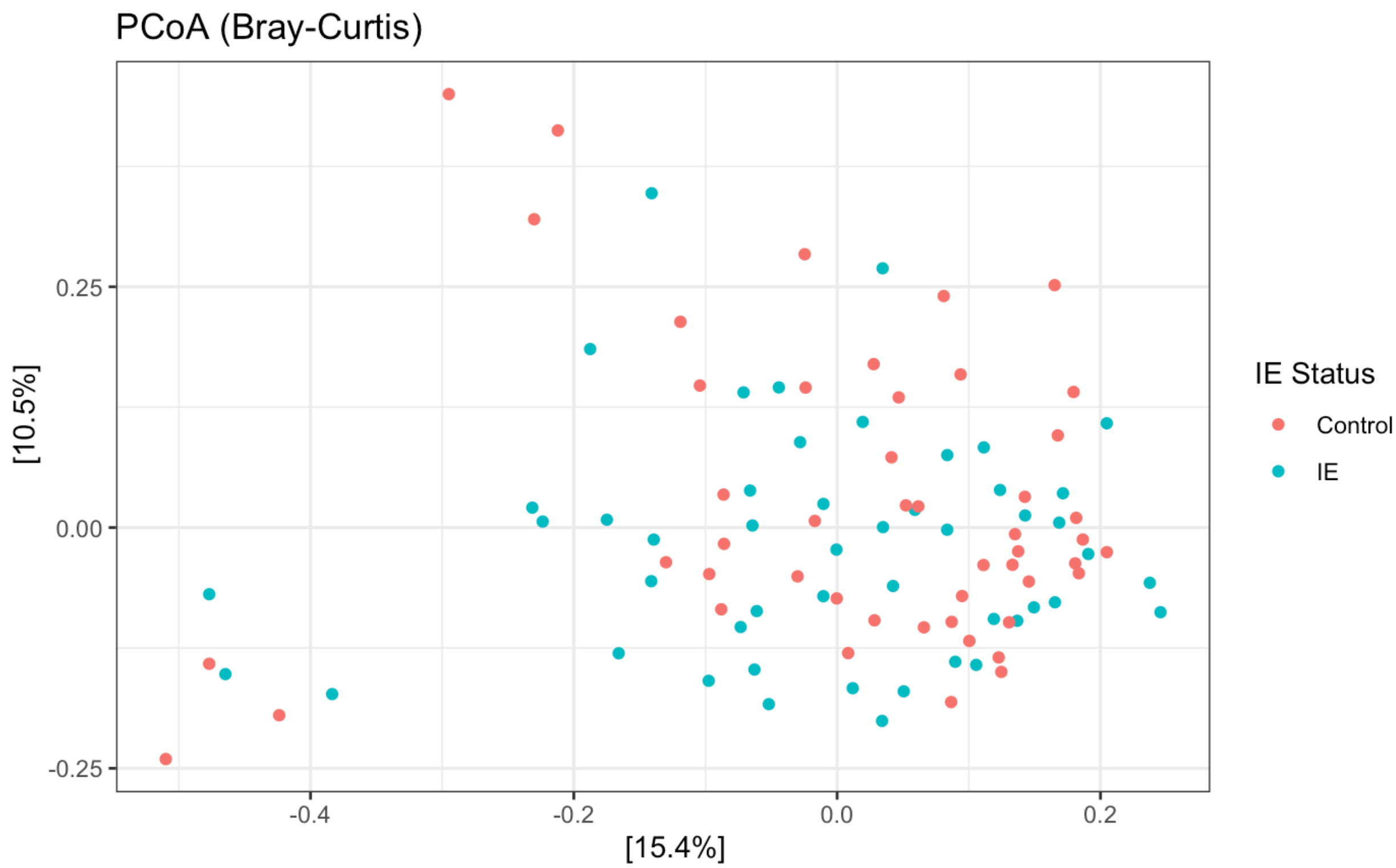

Figure S3: Principal coordinate analysis (PCoA) of gut microbial community composition based on Bray-Curtis dissimilarity. Each point represents one dog, colored by IE status.

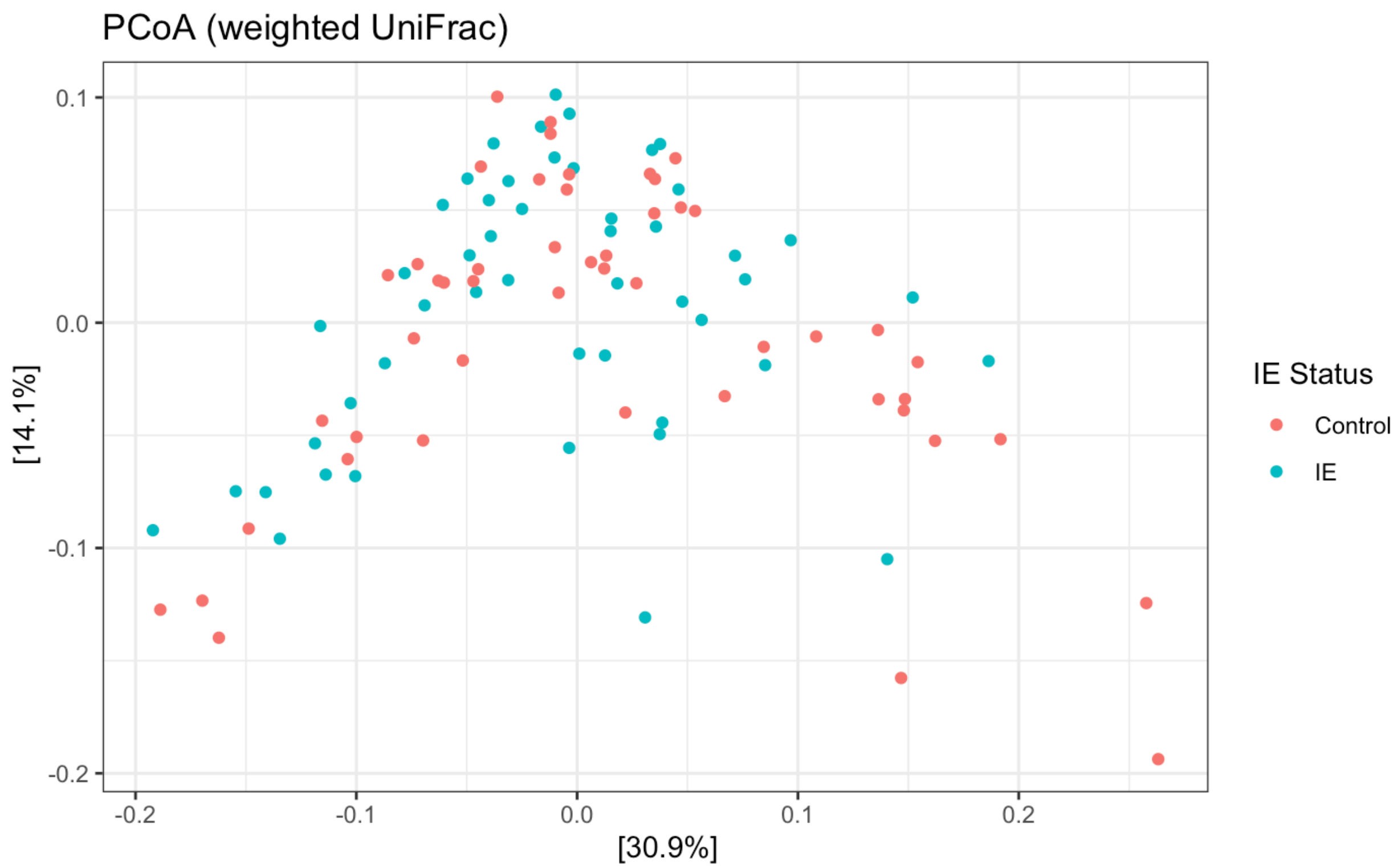

Figure S4: Principal coordinate analysis (PCoA) of gut microbial community composition based on weighted UniFrac dissimilarity. Each point represents one dog, colored by IE status.

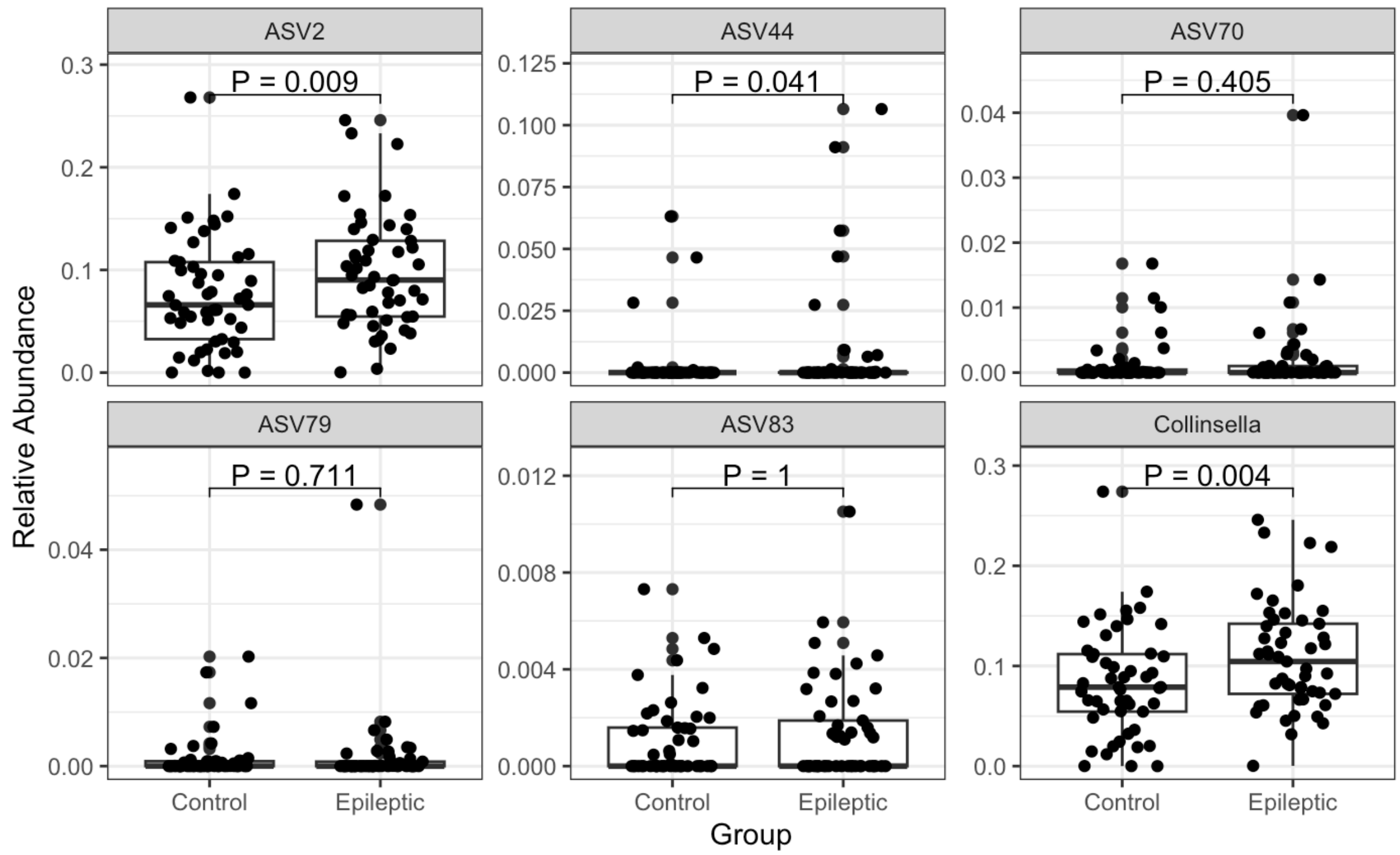

Figure S5: Relative abundance of *Collinsella* ASVs and total *Collinsella* in IE and control dogs. Boxplots show the distribution of relative abundance for each *Collinsella* ASV and total *Collinsella* genus. Each point represents one dog. P values are from pairwise sign test.
